# Acetylenic Metabolites and a 2-Phenoxychromone Derivative from the Aerial Parts of *Artemisia biennis*

**DOI:** 10.5812/ijpr-160050

**Published:** 2025-04-16

**Authors:** Maryam Najafi, Yalda Shokoohinia, Mahdi Mojarrab

**Affiliations:** 1 Students Research Committee, Kermanshah University of Medical Sciences, Kermanshah, Iran; 2 Ric Scalzo Institute for Botanical Research, Sonoran University of Medical Sciences, Tempe, USA; 3 Pharmaceutical Sciences Research Center, Research Institute for Health, Kermanshah University of Medical Sciences, Kermanshah, Iran

**Keywords:** *Artemisia biennis*, Flavonoid, Spiroketal-Enol Ethers, Cytotoxicity, Antifungal, Chemotaxonomy

## Abstract

**Background:**

* Artemisia biennis* is a weed of several crops, but some valuable biological effects have been reported from its various extracts.

**Objectives:**

The present phytochemical study was carried out on the dichloromethane (DCM) extract of the aerial parts of *A. biennis*. The prominent cytotoxic and antifungal activities of this extract were previously reported.

**Methods:**

The DCM extract was fractionated using vacuum-liquid chromatography (VLC). The selected fractions were purified by VLC and semi-preparative high-performance liquid chromatography (HPLC). The structures of the isolated compounds were characterized by comprehensive spectroscopic analyses.

**Results:**

Two acetylenic metabolites [(E)-en-yn-dicycloether, (Z)-en-yn-dicycloether], and a 2-phenoxychromone derivative (6-demethoxy-4'-O-methylcapillarisin) were isolated from the DCM extract of *A. biennis* aerial parts.

**Conclusions:**

These two acetylenic metabolites have exhibited various effects on biological systems. The results of this study are in accordance with the reported cytotoxic and antifungal activities of the DCM extract of *A. biennis*. The chemotaxonomic significance of the isolated compounds is also notable.

## 1. Background

The genus Artemisia (Asteraceae) comprises approximately 500 species (1). *Artemisia biennis* Willd. is one of the thirty-four Artemisia species growing in Iran (2). The dichloromethane (DCM) extract of *A. biennis* has been effective against K562 (IC_50_ = 64.86 µg/mL) and HL-60 (IC_50_ = 54.31 µg/mL) cancer cell lines and *Trichophyton verrucosum* (IC_50_ = 156.25 µg/mL) in cytotoxic and antifungal assays, respectively (3, 4). Previous phytochemical studies conducted on the volatile oil and hydroethanolic extract of the plant species resulted in the isolation and identification of some terpenoids, acetylenes, and flavonoids, respectively (5-8).

## 2. Objectives

The present phytochemical study was conducted on the DCM extract of the aerial parts of *A. biennis*. This extract was previously reported as a potent sample in cytotoxic and antifungal assays.

## 3. Methods

### 3.1. General Experimental Procedures

The NMR spectra were recorded on a Bruker Avance E (500 MHz) in CDCl_3_ and CD_3_OD as the solvents, with the residual solvent signal used as an internal standard. Electron impact mass spectrometry was performed on a 5973 Network Mass Selective Detector (Agilent). Separation was conducted on a semi-preparative high-performance liquid chromatography (HPLC) (Young Lin) equipped with a binary pump (YL 911S) and a PDA detector (YL 9160) using a Eurospher II 100-10 Si (250 × 20 mm ID, 10 μm) column.

### 3.2. Plant Materials

In the present study, aerial parts of *A. biennis* Willd. were collected from Zoshk, Razavi Khorasan, Iran.

### 3.3. Extraction and Isolation Procedures

**Figure 1.**
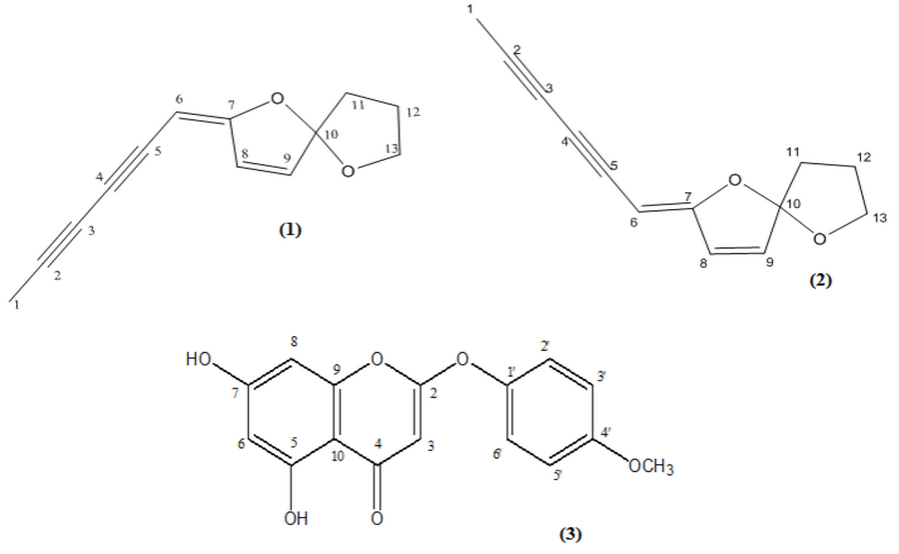
Chemical structures of isolated compounds (1 -3) from the dichloromethane (DCM) extract of *Artemisia biennis* aerial parts

Dried and powdered aerial parts of *A. biennis* (350.0 g) were extracted successively with petroleum ether (bp 40 - 60°C) (PE) and DCM by maceration at room temperature. The resultant extracts were filtered, and the filtrate was evaporated under reduced pressure to yield a crude extract. A large portion (23.6 g) of the crude DCM extract was suspended in methanol (MeOH) and kept at -20°C for 24 hours to eliminate long-chain fats. Then, fractionation of the extract (18.5 g) by vacuum liquid chromatography on a silica gel column (heptane/ethyl acetate (EtOAc), 8:2 → 0:10 and MeOH/EtOAc, 2:8 → 10:0) yielded 12 fractions (Fr. 1 - 12). A portion (440.0 mg) of Fr. 1 was purified using semi-preparative HPLC (hexane/EtOAc, 8:2, flow rate: 10 mL/min) to give a mixture of 1 and 2 (t_R_ = 12.0 min, 46 mg). A portion (320.0 mg) of Fr. 2 was purified using semi-preparative HPLC (PE/EtOAc, 8:2, flow rate: 10 mL/min) to afford 1 (t_R_ = 13.0 min, 4.5 mg) and 2 (t_R_ = 17.0 min, 21.7 mg) (Figure 1). A large portion (1.0 g) of Fr. 3 was fractionated by reversed-phase vacuum liquid chromatography using a gradient solvent system (A: MeOH/H_2_O (6:4), B: MeOH/H_2_O (8:2), C: MeOH/H_2_O (10:0), and D: Acetone). Crystallization of Fr. 3B from diethyl ether afforded 51.6 mg of 3 (Figure 1). 

## 4. Results

The present study resulted in the isolation of three components (1 - 3) from the DCM extract of *A. biennis* aerial parts (Figure 1). The chemical structures of the isolated compounds were identified as (1) (E)-en-yn-dicycloether, (2) (Z)-en-yn-dicycloether (9, 10), and (3) 6-demethoxy-4'-O-methylcapillarisin (11) by comparing their NMR and mass spectroscopic data with respective published data (9-11).

- Compound 1: (E)-En-yn-dicycloether; C_13_H_12_O_2_; spectroscopic data are presented in Appendix 1 in Supplementary File.

- Compound 2: (Z)-En-yn-dicycloether; C_13_H_12_O_2_; spectroscopic data are presented in Appendix 1 in Supplementary File.

- Compound 3: 6-Demethoxy-4'-O-methylcapillarisin; C_16_H_12_O_6_; spectroscopic data are presented in Appendix 1 in Supplementary File.

## 5. Discussion

The cytotoxic activity of the Artemisia species has attracted considerable attention in recent years. The majority of isolated active components are terpenoids, but other types of secondary metabolites, such as flavonoids and coumarins, also play a significant role (12). The DCM extract of *A. biennis* has shown potent cytotoxicity against cancerous cell lines (3). The present study reports the isolation of two spiroketal-enol ethers (1-2) and a flavonoid (3) from the DCM extract of *A. biennis*. Compound 1 has shown spasmolytic, antiphlogistic (13), and anti-inflammatory activities (14). It interferes with the LPS-induced production of IL-1, IL-6, TNF, and PGE2 in primary human monocytes (14). The anti-feeding activity of compound 2 against *Spodoptera littoralis*, *Myzus persicae*, and *Pieris brassicae* has been reported (15, 16). Both acetylenic metabolites exhibited antibacterial (against *Staphylococcus aureus* and *Shigella sonnei*), insecticidal (against *Culex quinquefasciatus*), cytotoxic (against A549, B16F1, and SK-Mel-2 cell lines), and antifungal (against *Trichophyton mentagrophytes*) activities (16-18). In addition, *Bacillus subtilis* is susceptible to compound 1 (17). There is no reported biological activity for compound 3. Interestingly, capillarisin isolated from *A. capillaris* has shown an antiproliferative effect on prostate carcinoma cells (19).

Compounds 1 and 2 have been previously identified in the essential oil of *A. biennis* from the west of Canada and northeast of Iran (6, 7). However, in another study, none of the isomers were identified in the essential oil of *A. biennis* from Iran (5). Despite the inconsistency of different floras in terms of the growth of this species in Iran (2), there seems to be a close similarity between the profile of secondary metabolites — and bioactivities against dermatophytes — of Iranian and Canadian plant samples (4, 6, 7). The spiroketal enol ethers can be regarded as characteristic chemical markers of the Anthemideae tribe (9). They have also been used for infrageneric subdivisions in the genus Artemisia (20). 6-Demethoxy-4'-O-methylcapillarisin has a 2-phenoxychromone structure, which is an uncommon class of flavonoids and has an unusual bond between rings B and C (21). 2-Phenoxychromones are rare compounds as secondary metabolites of higher plants and are thus clear chemotaxonomic indexes (22).

### 5.1. Conclusions

The cytotoxic and antifungal activity reported from the DCM extract of *A. biennis* is likely associated with the presence of acetylenic metabolites [(E)-en-yn-dicycloether, (Z)-en-yn-dicycloether]. Additionally, the isolated compounds in the current study may serve as potential chemotaxonomic markers for *A. biennis*.

ijpr-24-1-160050-s001.pdf

## Data Availability

The data presented in this study are uploaded during submission as a supplementary file and are openly available for readers upon request.
