# Supplementary material for: Acetylenic Metabolites and a 2-Phenoxychromone Derivative from the Aerial Parts of Artemisia biennis
Source: Iran J Pharm Res. 2025 Apr 16;24(1):e160050. doi: 10.5812/ijpr-160050 (PMC12285677; doi:10.5812/ijpr-160050)

Appendix 1. Acetylenic metabolites and a 2-phenoxychromone derivative from the aerial parts of *Artemisia biennis*

Compound **1**: (E)-En-yn-dicycloether (**1**) $C_{13}H_{12}O_2$; EI-MS (m/z): 200.1 $[M]^{+}$. ^{1}H NMR (500 MHz, $CDCl_3$, *J* in Hz): δH 6.70 (d, 5.7, H-8), 6.19 (dd, 5.7, 1.7, H-9), 4.90 (brs, H-6), 3.90- 4.20 (m, CH_2–13), 2.05- 2.25 (m, CH_2–11, CH_2–12), 1.97 (d, 1, CH_3-1). ^{13}C NMR (125 MHz, $CDCl_3$): δ C 168.8 (C-7), 135.7 (C-9), 126.0 (C-8), 120.8 (C-10), 79.9 (C-6), 79.6 (C-2), 76.3 (C-4), 71.5 (C-5), 69.7 (C-13), 64.9 (C-3), 35.5 (C-11), 24.5 (C-12), 4.7 (C-1).

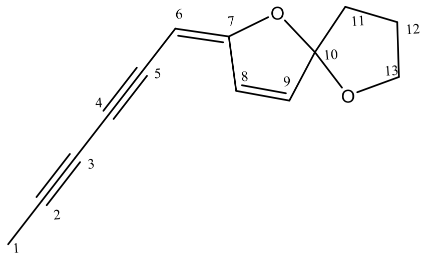

Compound **2:** (Z)-en-yn-dicycloether (**2**) $C_{13}H_{12}O_2$; EI-MS (m/z): 200.1 $[M]^{+}$. ^{1}H NMR (500 MHz, $CDCl_3$, *J* in Hz): δ H 6.23 (d, 5.6, H-8), 6.15 (d, 5.6, H-9), 4.59 (brs, H-6), 3.98- 4.28 (m, CH_2–13), 2.06- 2.33 (m, CH_2–12), 2.06- 2.22 (m, CH_2–11), 1.98 (d, 1, CH_3-1). ^{13}C NMR (125 MHz, $CDCl_3$): δ C 167.1 (C-7), 135.2 (C-9), 127.4 (C-8), 121.0 (C-10), 80.6 (C-2), 78.9 (C-4), 78.8 (C-6), 70.7 (C-5), 69.7 (C-13), 65.1 (C-3), 35.6 (C-11), 24.5 (C-12), 4.7 (C-1).

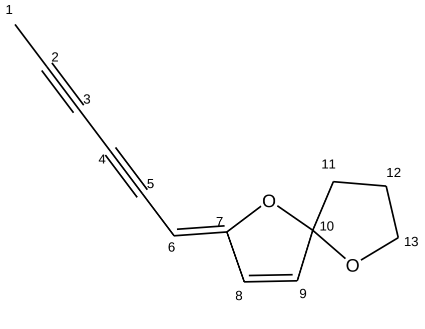

Compound **3**: 6-demethoxy-4'-O-methylcapillarisin (**3**) $C_{16}H_{12}O_6$; EI-MS (m/z): 300.1 $[M]^{+}$. ^{1}H NMR (400 MHz, CD_3OD, *J* in Hz): δ H 7.21 (d, 9.2, H-2’, H-6’), 7.05 (d, 9.2, H-3’, H-5’), 6.31 (d, 2.2, H-8), 6.20 (d, 2.2, H-6), 5.12 (s, H-3), 3.84 (s, $-OCH_3$). ^{13}C NMR (125 MHz, $CDCl_3$): δ C 183.9 (C-4), 168.5(C-2), 164.4 (C-7), 161.5 (C-5), 158.6 (C-4’), 155.4 (C-9), 145.1 (C-1’), 121.5 (C-2’, C-6’), 115.0 (C-3’, C-5’), 102.2 (C-10),99.2 (C-6),93.6 (C-8), 86.7 (C-3), 54.8 ($-OCH_3$).

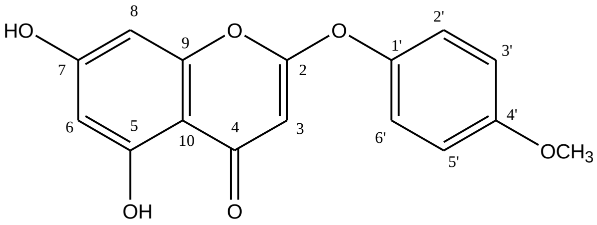

Supplement: ijpr-24-1-160050-s001.pdf [file ijpr-24-1-160050-s001.pdf]
